# Hypomagnesemia, insulin secretion and action in patients without diabetes, 1 year after kidney transplantation

**DOI:** 10.3389/fmed.2025.1492871

**Published:** 2025-01-22

**Authors:** Rasmus K. Carlsen, Anders Åsberg, My Svensson, Kåre I. Birkeland, Hanne S. Jørgensen, Iain Bressendorff, Hanne L. Gulseth, Karsten Midtvedt, Espen Nordheim, Trond G. Jenssen

**Affiliations:** ^1^Department of Transplantation Medicine, Oslo University Hospital and University of Oslo, Oslo, Norway; ^2^Department of Pharmacy, University of Oslo, Oslo, Norway; ^3^Department of Nephrology, Akershus University Hospital, Lørenskog, Norway; ^4^Department of Nephrology, Aalborg University Hospital, Aalborg, Denmark; ^5^Institute of Clinical Medicine, University of Oslo, Oslo, Norway; ^6^Department of Kidney Disease, Aarhus University Hospital, Aarhus, Denmark; ^7^Institute of Clinical Medicine, Aarhus University, Aarhus, Denmark; ^8^Department of Nephrology, Herlev and Gentofte Hospital, Herlev, Denmark; ^9^Division of Mental and Physical Health, Norwegian Institute of Public Health, Oslo, Norway; ^10^Department of Nephrology, Ullevål Hospital, Oslo University Hospital, Oslo, Norway

**Keywords:** hypomagnesemia, kidney transplantation, post-transplant diabetes mellitus, insulin secretion, insulin action, oral glucose tolerance test

## Abstract

**Introduction:**

Hypomagnesemia after kidney transplantation has been reported as a potential risk factor for development of post-transplant diabetes mellitus.

**Methods:**

In kidney transplant recipients undergoing an oral glucose tolerance test during one-year surveillance follow-up we estimated insulin sensitivity with the Matsuda index, a modified Stumvoll index, and HOMA-2_IR_. First and second phase insulin secretion was assessed using the Stumvoll equation. Participants were categorized into tertiles by plasma magnesium levels, (<0.7, 07–0.78,>0.78 mmol/L).

**Results:**

We included 208 patients (62% men, median age 51 years). Patients in the lowest compared to the highest magnesium tertile had higher measured GFR (mean 59 vs. 49 mL/min, *p* = 0.002), tacrolimus trough concentration (mean 6.7 vs. 5.5 μg/L, *p* < 0.001), and fasting plasma glucose (mean 5.5 vs. 5.3 mmol/L, *p* = 0.03). There was no significant difference in the Matsuda index between magnesium tertiles, nor in insulin sensitivity assessed by modified Stumvoll index, HOMA-2_IR_, first or second phase insulin. Results indicate a non-significant trend toward lower disposition index in the lowest vs. highest tertile (*p* = 0.052).

**Conclusion:**

In kidney transplant recipients with lower compared to normal plasma magnesium levels we found a higher fasting plasma glucose but no differences in insulin sensitivity indexes nor dynamic insulin measurements.

## Introduction

Despite recovery of kidney function, kidney transplant recipients have an annual death rate approximately twice as high as the general population, mainly due to cardiovascular disease and associated risk factors such as diabetes, hypertension, and dyslipidemia (1).

Post-transplant diabetes mellitus (PTDM) and impaired glucose tolerance (IGT) are major risk factors for cardiovascular events in kidney transplant recipients, and prevention of PTDM is therefore a major concern for health caretakers (2–4). The cumulative incidence of PTDM is estimated to be between 13 and 29% 1 year after engraftment (2, 5, 6). The high incidence of PTDM is to a large degree related to the use of immunosuppressant drugs such as steroids and calcineurin inhibitors, but post-transplant hypomagnesemia has also been suggested to be a risk factor for development of hyperglycemia after kidney transplantation (6).

Magnesium is an important cofactor for a number of cellular functions related to insulin action and secretion, e.g., for the normal function of the insulin receptor, and it has been postulated that hypomagnesemia may contribute to insulin resistance (7). Indeed, hypomagnesemia is associated with increased insulin resistance in patients with type 2 diabetes, and magnesium supplementation may decrease insulin resistance in patients with a high risk of diabetes (8–10). Some studies also indicate that low serum magnesium may be associated with impaired insulin secretion (11, 12). In addition, short-term intervention with oral magnesium supplementation decreases fasting plasma glucose in pre-diabetic and diabetic individuals (10).

The high prevalence of hypomagnesemia after kidney transplantation is mainly caused by renal magnesium wasting linked to the use of immunosuppressive drugs, i.e., from calcineurin inhibitors, with impaired tubular reabsorption of magnesium (13, 14). In addition, the use of loop diuretics, inadequate intake of magnesium, as well as decreased intestinal absorption of magnesium may also contribute to low magnesium levels (14). Two recent cohort studies have addressed the relationship between serum magnesium and mortality in kidney transplant recipients (15, 16). While both studies found an increased mortality rate in patients with high magnesium, the results for patients with low magnesium were conflicting. In the first study, low magnesium was associated with a decreased mortality rate (15), while the second study found an increased mortality rate in patients with low magnesium (16).

Post-transplant hypomagnesemia has been reported as a potential risk factor for PTDM in kidney transplant recipients (6, 17). Two randomized controlled trials from the same research group investigated the effect of magnesium supplementation on kidney transplant recipients 2 weeks and 4 years after transplantation, respectively (18, 19). In one of these studies, the results were inconclusive, as only a limited effect was observed on fasting glucose (18), whereas in the other study, no difference was seen in first-phase insulin response, fasting glucose, HbA1c, or insulin resistance (19).

In the present study we hypothesized that hypomagnesemia in patients without known diabetes would be associated with lower insulin sensitivity and impaired insulin response to an oral glucose tolerance test (OGTT) 1 year after kidney transplantation.

## Materials and methods

### Study design and inclusion

This was an observational, cross-sectional study with recruitment taking place between February 28th, 2019, and March 1st, 2021. Eligible participants were patients undergoing one-year planned routine follow-up after kidney-only transplantation at our department. We included patients with available measurements of fasting plasma magnesium and glucose and insulin measurements during an oral glucose test (OGTT). Patients with pharmacologically treated diabetes at the time of examination did not undergo OGTT and were not included in this analysis. The study was approved by the Regional Committee for Medical and Health Research Ethics, South-East (2014/455 REK sør-øst B). All study participants had signed a written informed consent.

### Laboratory data

After fasting overnight, the patients underwent an OGTT with plasma glucose and insulin measured at 0, 30, and 120 min after intake of 75 g glucose dissolved in 200 mL water. Plasma glucose was analyzed after centrifugation using a glucose dehydrogenase method (Cobas 6,000, Hitachi, Roche, Rotkreuz, Switzerland). Plasma insulin was measured using electrochemical luminescence immunoassay (Cobas e602, Rotkreuz, Switzerland). Plasma magnesium was analyzed in heparinized blood using a colorimetric endpoint assay (Cobas 6,000, Hitachi, Roche, Rotkreuz, Switzerland). Measured glomerular filtration rate (mGFR) was determined using 2-point iohexol serum clearance (20).

### Glucose and insulin indexes

Total area under the curve (AUC) for glucose and insulin was calculated as integrals using 0-, 30-, and 120-min values by the trapezoidal rule.

Insulin sensitivity was estimated by the Matsuda insulin sensitivity index (21) using average concentration values of glucose and insulin calculated as AUC_0-120_/120 min (22).

Insulin sensitivity was also assessed by the modified Stumvoll insulin sensitivity index (23).

First and second phase insulin release was calculated according to the equations of Stumvoll et al. (24):

*First phase insulin = 1,283 + 1.829 × Insulin_30_-138.7 × Glucose_30_ + 3.772 × Insulin_0_.*

*Second phase insulin = 286 + 0.416 × Insulin_30_-25.94 × Glucose_30_ + 0.926 × Insulin_0_.*

Homeostatis Model Assessment of insulin resistance (HOMA-2_IR_) and of beta cell function (HOMA-2_B%_) was calculated using the online available calculator (25).

Insulinogenic index was calculated using the formula as suggested elsewhere by Pacini et al. (26):

*Insulinogenic index = (Insulin_30_ – Insulin_0_) / (Glucose_30_ – Glucose_0_).*

Disposition index was calculated using the formula (27, 28):

*Disposition index = Matsuda index × first phase insulin secretion.*

*Insulin index = 0.208–0.0032 × BMI – 0.0000645 × Ins_120_–0.00375 x Gluc_120_.*

BMI is body mass index calculated as BMI = weight/height^2^.

PTDM, impaired fasting glucose (IFG), and impaired glucose tolerance (IGT) were defined using the American Diabetes Association’s classifications (29) in accordance with international consensus (30): PTDM was defined as fasting glucose ≥7 mmol/L or 2-h glucose ≥11.1 mmol/L and no previously known diabetes, i.e., no pretransplant diabetes and no diabetes at our routine visit with OGTT 8 weeks after transplantation. IFG was defined as fasting glucose 5.6–6.9 mmol/L. IGT was defined as 2-h glucose 7.8–11.0 mmol/L.

### Statistical analysis

Data were analyzed using Stata 16 for Windows (StataCorp LP, College State, TX). Patient characteristics are presented as mean ± standard deviation or median (range) if data were skewed. Categorical data are presented as numbers (percentage). Distributions were tested with histograms and QQ plots.

Differences between magnesium tertiles were tested with one-way analysis of variance (ANOVA) and equal standard deviation between groups using Bartlett’s test. If continuous variables were not normally distributed, we used logarithmic transformation. If normal distribution was not achieved, we used Kruskal-Wallis. Student’s t-test were used for between-group differences. Wilcoxon-Mann–Whitney test were used if data were skewed, even after logarithmic transformation. Categorical data were compared with chi-square test. Sensitivity analysis was performed post-hoc using univariable and multivariable linear regression with predefined variables: age, gender, triglycerides, plasma creatinine and trough tacrolimus.

Sample size calculation was based on a clinically meaningful difference in the Matsuda index of 1.5 between lowest and highest magnesium tertile. With a power of 80%, a significance level of 5%, and an expected standard deviation of 2.8 (31), the study would require 55 participants in each tertile for a total of 165 participants.

## Results

### Study population

**Figure 1:**
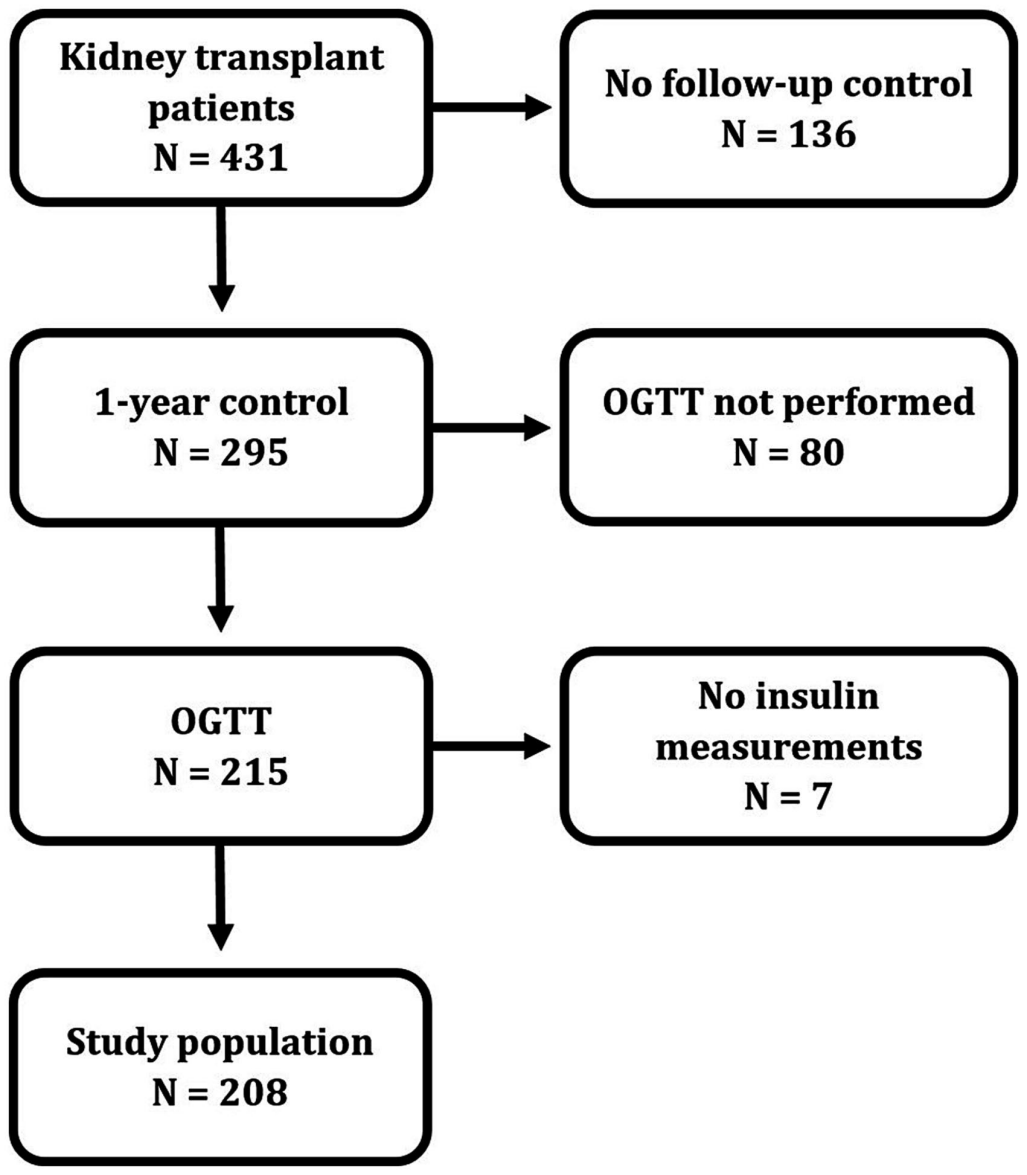
Flow chart of patient inclusion.

From February 28th, 2019 till January 31st, 2021, a total of 431 adult patients were eligible for the standard surveillance follow-up 1 year after kidney transplantation (Figure 1). Of these, 136 patients did not participate in the 1-year routine follow-up. Six of these patients had died, and five had graft loss prior to the one-year control. Furthermore, 73 patients did not show up due to COVID-related restrictions, five patients because of other medical reasons, six patients did not consent, and 41 patients did not attend for unknown reasons.

Of the remaining 295 patients performing the one-year investigation, eighty patients did not undergo an OGTT for the following reasons: Sixty because they had known diabetes, 11 because of other medical conditions, three because of time limitations, four because they carried an infectious disease and two for unknown reasons. Insulin was not measured in seven patients. The remaining 208 patients comprises the study population.

The 223 patients who were not included in this analysis were older than those included (median age 55 (range 20–82) years versus 51 (range 20–84) years, *p* = 0.002). There were no differences in proportion of men (69% versus 62% in the study population, *p* = 0.12), magnesium levels (mean 0.73 versus 0.74 mmol/L in the study population, *p* = 0.82) and median tacrolimus (6.3 versus 6.3 μg/L in the study population, *p* = 0.28). Likewise, cytomegalovirus (CMV) serostatus, COVID infection and/or hospitalization and acute rejections were comparable.

Participants were categorized into tertiles by plasma magnesium levels, (<0.7, 07–0.78,>0.78 mmol/L). Patient characteristics according to tertiles of plasma magnesium are presented in Table 1. Age, sex, and BMI were not significantly different across tertiles. Patients in the lowest tertile had a higher measured GFR and a higher tacrolimus concentration compared to those in the highest tertile. Furthermore, patients in the lowest magnesium tertile had lower total cholesterol concentrations and higher fasting plasma glucose than those in the highest tertile. Use of medications, including magnesium supplementation, were comparable across magnesium tertiles, with the exception of a higher prevalence of tacrolimus, as compared to ciclosporin, in the lowest versus the highest tertile. CMV serostatus and COVID infection and/or hospitalization were also comparable across magnesium tertiles.

**Table 1:** Patient characteristics according to tertiles of plasma magnesium 1 year after kidney transplantation.

	1st tertile	2nd tertile	3rd tertile	*p*-value
Patients (*n*)	74	71	63	
Age (years)	53 ± 15	48 ± 17	54 ± 15	0.10
Male	47 (64%)	46 (65%)	36 (57%)	0.63^a^
BMI (kg/m^2^)	25 ± 4	25 ± 4	25 ± 4	0.94
Number of previous transplantations	0 [0–4]	0 [0–2]	0 [0–2]	0.77^b^
Prednisolone dose, mg	5 [5–20]	5 [5–10]	5 [5–7.5]	0.91^b^
Chronic kidney disease status - baseline				
Creatinine, μmol/L	101 [57–249]	110 [49–282]	130 [52–277]	0.007^b^
mGFR (ml/min/1.73 m^2^)	59 ± 16	57 ± 15	49 ± 21	0.003
Biochemistry				
Magnesium, mmol/L	0.67 [0.47–0.7]	0.74 [0.71–0.77]	0.82 [0.78–1.1]	<0.001
Tacrolimus trough concentration^c^, μg/L	6.7 ± 1.7	6.3 ± 1.5	5.5 ± 1.3	<0.001
ALAT, U/L	18 [5–133]	20 [8–63]	20 [7–53]	0.25
CRP, mg/L	1.6 [0.6–37]	1.5 [0.6–7.8]	1.2 [0.6–25]	0.62
Vitamin D, nmol/L	63 [25–137]	67 [24–128]	66 [12–145]	0.67
Blood pressure				
Systolic blood pressure, mmHg	138 ± 17	137 ± 16	138 ± 17	0.81
Diastolic blood pressure, mmHg	78 ± 10	78 ± 11	78 ± 11	0.90
Glucose metabolism				
Fasting blood glucose, mmol/L	5.5 ± 0.6*	5.6 ± 0.8	5.3 ± 0.6	0.04
30 min glucose, mmol/L	8.2 ± 1.5	8.2 ± 2.0	7.9 ± 1.6	0.48
2-h glucose, mmol/L	6.6 ± 1.8	6.4 ± 1.7	6.1 ± 1.8	0.33
Fasting insulin, pmol/L	83 [21–325]	83 [29–407]	76 [20–476]	0.35
30 min insulin, pmol/L	435 [9–1,488]	441 [52–1,665]	458 [166–1,178]	0.86^b^
2-h insulin, pmol/L	288 [51–1838]	277 [32–767]	270 [21–1,061]	0.29
HbA1c, % (mmol/mol)	5.7 ± 0.5 (39 ± 5)	5.6 ± 0.5 (38 ± 5)	5.7 ± 0.3 (38 ± 4)	0.73
Lipid metabolism (fasting)				
Triglycerides, mmol/L	1.3 [0.6–3.4]	1.3 [0.6–7.8]	1.6 [0.6–8.3]	0.052
Total cholesterol, mmol/L	4.4 [2.8–6.5]*	4.4 [2.7–7.1]	4.7 [2.1–11]	0.04^b^
HDL, mmol/L	1.4 [0.9–3.5]	1.4 [0.5–3.1]	1.5 [0.6–2.9]	0.43
LDL, mmol/L	2.6 [0.8–4.7]	2.6 [1.2–5.4]	2.8 [1.4–5.5]	0.29
Lipoprotein A, nmol/L	18 [7–393]	13 [7–260]	16 [7–296]	0.25^b^
Medication				
Tacrolimus	71 (96%)*	70 (99%)	51 (81%)	<0.001^a^
Mycophenolate mofetil	69 (93%)	67 (94%)	59 (94%)	0.96^a^
Ciclosporine	2 (3%)*	1 (1%)	9 (14%)	0.02^a^
Statin	48 (65%)	49 (69%)	40 (63%)	0.78^a^
Proton pump inhibitor	47 (64%)	38 (54%)	35 (56%)	0.44^a^
Loop diuretics	3 (4%)	11 (15%)	8 (13%)	0.07^a^
Thiazide	1 (1%)	0 (0%)	0 (0%)	0.40^a^
Magnesium supplement	15 (20%)	13 (18%)	6 (10%)	0.20^a^

BMI, body mass index; mGFR, measured glomerular filtration rate; ALAT, alanine aminotransferase; CRP, C-reactive protein; Vitamin D, 25-hydroxyvitamin D. Data are presented as mean ± standard deviation if normally distributed or median [range] if skewed. Categorical data are presented as number (%). *p*-values are differences between tertiles using oneway ANOVA, a, chi-square test for categorical data; b, Kruskal Wallis ranksum test if data were not normally distributed even after logarithmic transformation; c, Excluded patients not on tacrolimus (*n* = 16). *Difference between highest and lowest magnesium tertile, *p* < 0.05.

### Insulin and glucose outcomes

Insulin and glucose indexes are presented in Table 2. There was no significant difference in insulin sensitivity between magnesium tertiles, neither assessed by the Matsuda index, nor by other measures of insulin sensitivity, like the modified Stumvoll index or HOMA-2_IR_ index. Likewise, there were no differences between tertiles in first and second phase insulin, AUC of insulin or glucose, insulinogenic index, or HOMA-2_B%_. However, the disposition index was significantly different between magnesium tertiles (*p* = 0.049), with a non-significant trend toward a lower index in the lowest tertile compared with the highest (*p* = 0.052). Sensitivity analysis with linear regression did not show any association between magnesium values and any outcome in neither univariable nor multivariable regression (*p* ≥ 0.19). All linear regressions models had poor fit in unadjusted models with *R*^2^ < 0.01.

**Table 2:** Insulin and glucose indexes according to tertiles of magnesium after 1 year.

	1st tertile*	2nd tertile	3rd tertile	*p*-value
Patients (*n*)	74	71	63	
Matsuda	3.1 [0.7–11.4]	3.1 [0.9–9.4]	3.6 [0.7–8.0]	0.45
AUC-Insulin, pmol/l *×* min	329 [78–1,306]	355 [66–1,051]	340 [139–858]	0.77
AUC-Glucose, mmol/l *×* min	7.2 ± 1.3	7.2 ± 1.4	6.9 ± 1.2	0.35
Insulinogenic index, pmol/mmol	117 [−1,180–838]	145 [−635–2,595]	144 [−1,633–536]	0.75^a^
HOMA2 _B%_	106 [50–348]	114 [52–262]	113 [46–278]	0.80
HOMA2_IR_	1.6 [0.4–6.9]	1.7 [0.4–8.7]	1.5 [0.6–7.6]	0.84
First-phase insulin release, pmol/l	1,342 [221–3,051]	1,381 [263–4,962]	1,344 [504–3,463]	0.90^a^
Second-phase insulin release, pmol/l	340 [94–751]	359 [124–1,187]	354 [160–825]	0.85
Disposition index	3,956 [1225–9,214]	4,189 [1012–10,461]	4,965 [1759–10,112]	0.049
Insulin index, modified Stumvoll, ml/min/kg	0.085[−0.038–0.124]	0.082 [0.032–0.128]	0.086 [0.009–0.125]	0.78^a^

AUC, area under the curve; HOMA2_IR,_ Homeostatis Model Assessment for Insulin Resistance; HOMA2_B%_, Homeostatis Model Assessment for Beta Cell function. Data are presented as mean ± standard deviation if normally distributed or median [range] if skewed. *Magnesium tertiles: 1st ≤ 0.71; 2nd 0.71–0.77; 3rd ≥ 0.78 mmoL/L. *p*-values are differences between tertiles using oneway ANOVA or a: Kruskal Wallis ranksum test if data were not normally distributed even after logarithmic transformation.

### Hyperglycemic classifications

There were no differences in hyperglycemic classifications according to magnesium tertiles. Eight patients had glucose measurements that would qualify for a diagnosis of diabetes: one in the lowest, five in the middle and two in the highest tertile of magnesium. Thirty-nine patients had impaired glucose tolerance; 18 (24%) in the lowest, 11 (15%) in the middle and 10 (16%) in the highest tertile of magnesium. The presence of impaired glucose tolerance was not significantly different between the lowest and the highest magnesium tertile (*p* = 0.22) in patients without PTDM. Sixty-seven patients had impaired fasting glucose at the one-year control; 28 (38%) in the lowest, 24 (34%) in the middle and 15 (24%) in the highest magnesium tertile (*p* = 0.20).

## Discussion

In this study, we investigated the link between magnesium concentrations and parameters of insulin resistance and secretion in kidney transplant recipients without pharmacological treated or known diabetes 1 year after transplantation. Although we found a slightly higher fasting plasma glucose and lower disposition index in subjects with lower plasma magnesium, we found no difference in insulin sensitivity and secretion in different magnesium tertiles. The lack of association did not change when we assessed insulin sensitivity using Matsuda index, modified Stumvoll index or HOMA-2_IR_.

We hypothesized that patients with lower plasma magnesium would have higher insulin resistance, as the tyrosine kinase in the insulin receptor uses magnesium as a cofactor with adenosine-tri-phosphate (7). Our hypothesis was, however, not confirmed. This may seem inconsistent with findings in a meta-analysis in non-transplanted patients with high risk of diabetes, which found an improvement of HOMA-2_IR_ after short-term intervention of magnesium supplementation (10). However, our results are in concordance with treatment studies in kidney transplant recipients (19) and a meta-analysis of placebo-controlled randomized clinical trials in patients with diabetes (10).

Several previous studies found an association between hypomagnesemia and PTDM in kidney transplant recipients (6, 17, 32), but to our knowledge, only the paper by Van Laecke et al. (19) addressed the relationship between hypomagnesemia and insulin concentrations in such patients. In their study, which included patients from 4 months onwards (median 4 years) after kidney transplantation, neither insulin release nor HOMA-2_IR_ assessed insulin resistance changed in patients randomized to oral magnesium supplementation for 6 months (19).

The pathogenesis of type 2 diabetes and PTDM may not be identical. The American Diabetes Association classifies PTDM as an “other specific type” of diabetes, indicating that PTDM is a different type than for example type 2 diabetes mellitus (33). Whereas patients with PTDM also exhibit characteristics such as increased insulin resistance (23), lower insulin release, obesity and high levels of triglycerides (34), there are differences in time of diagnosis and progression of diabetes, use of immunosuppressive agents, incidence of viral infections and magnesium levels (35). PTDM has a bimodal incidence curve with a first peak occurring approximately 3 months after transplantation, followed by a decline and then a slow increase in incidence from about 1 year after transplantation and onwards (36). The first wave could primarily be related to high doses of corticosteroids and calcineurin inhibitors, while the second wave could have a significant contribution from conventional diabetogenic risk factors, e.g., age, weight, and genetic disposition. The role of magnesium, if any, in the different incidence waves of PTDM is not clear.

Patients in the lowest magnesium tertile had a higher fasting glucose compared to those in the highest tertile. This is in concordance with the findings in a meta-analysis of non-transplanted patients at risk for diabetes (10), while a much smaller study in kidney transplant patients found no such difference (19).

The pathway of hypomagnesemia leading to hyperglycemia may be due to both insulin action and insulin secretion. Important to insulin action, magnesium acts as a co-factor downstream to the insulin-receptor and also as a co-factor to enzymatic reactions related to glycolysis (37). Insulin resistance and hyperglycemia, however, may in itself also lead to hypomagnesemia because of urinary wasting from osmotic hyperglycuria, hyperfiltration and a downregulation of the magnesium transporter the transient receptor potential melastatin 6 (TRPM6) in the distal convoluted tubule, which leads to a decreased reabsorption of magnesium (13, 14, 38). An association between low magnesium levels and the function of TRPM6 and related transport proteins may also have an impact on insulin secretion. However, acute lowering of extracellular magnesium did not impair glucose-stimulated insulin secretion *in vitro* in mouse islets (39).

In our study, the prevalence of hypomagnesemia was considerable, which resulted in the lowest tertile of magnesium having the upper range of the normal cutoff at 0.7 mmol/L. Tacrolimus dose and trough levels were higher in patients in the lowest tertile of magnesium, which is consistent with the hypothesis that tacrolimus increases urinary excretion of magnesium. The mechanism of increased excretion, as with insulin resistance, is likely a downregulation of TRPM6, which leads to a decreased distal reabsorption of magnesium (14). Tacrolimus is primarily associated with development of PTDM through its calcineurin inhibitor effect in beta cells (40). Whether tacrolimus contributes to the development of PTDM in part through increased urinary excretion of magnesium remains speculative.

### Strengths and limitations

Strengths of our study include a substantial number of patients investigated at a standardized time-point post-transplant, the comprehensive investigations of glucose metabolism, and use of measured GFR. Using OGTT for assessment of prediabetes and PTDM is in concurrence with international consensus in kidney transplant patients (30). The excluded cohort was well defined regarding variables such as age, sex, tacrolimus trough levels and diabetes occurrence and only differed significantly in age on the compared parameters.

There are several limitations to this study. Despite only accounting for 0.3% of the body total magnesium (38), plasma or serum magnesium may not reflect the total amount of magnesium in the body, although it is the commonly available test to measure magnesium deficiency. Other quantifications of magnesium include ionized magnesium, intracellular magnesium in red blood cells, skeletal muscle, bone, peripheral lymphocytes and other cells, muscle or bone biopsies, and in urine, e.g., as magnesium tolerance test (41). Although a magnesium tolerance test is often described as the gold standard method for quantification of magnesium, none of the mentioned measurements have been validated with regards to clinical endpoints. Another shortcoming is that we did not have serial measurements of magnesium, glucose and insulin prior to the assessments. There may be an effect depending on time of exposure from low levels of magnesium, which because of the design could not be tested. We did not have data on diet or lifestyle which could impact our results.

We used the Matsuda insulin sensitivity index as a surrogate measure for insulin sensitivity and a modified Stumvoll sensitivity index. An earlier study in kidney transplant recipients showed a relatively poor correlation between these quantifications and insulin sensitivity index measured with a euglycemic-hyperinsulinemic glucose clamp with Spearman’s correlations of 0.41 and 0.58, respectively (23). It would have been optimal to assess insulin sensitivity by an euglycemic hyperglycemic clamp technique (42), but this is rarely feasible in larger studies like this. Lastly, we had a high dropout rate and especially patients with overt diabetes were excluded from performing an OGTT. Therefore, it could be that our cohort without known diabetes had so marginal differences in insulin resistance that we were not able to find them with surrogate measures for insulin sensitivity.

In conclusion, we found no differences in neither insulin sensitivity indexes nor dynamic insulin measurements across tertiles of plasma magnesium in kidney transplant recipients. We found higher fasting plasma glucose and a trend toward lower disposition index in recipients with low magnesium levels. Our results do not support an important role for hypomagnesemia on insulin sensitivity or development of PTDM after kidney transplantation.

## Data Availability

The raw data supporting the conclusions of this article will be made available by the authors, without undue reservation.

## References

[1] Foley RN Parfrey PS Sarnak MJ. Clinical epidemiology of cardiovascular disease in chronic renal disease. Am J Kidney Dis. (1998) 32:S112–9. doi: 10.1053/ajkd.1998.v32.pm9820470, PMID: 9820470

[2] Cosio FG Kudva Y van der Velde M Larson TS Textor SC Griffin MD. New onset hyperglycemia and diabetes are associated with increased cardiovascular risk after kidney transplantation. Kidney Int. (2005) 67:2415–21. doi: 10.1111/j.1523-1755.2005.00349.x, PMID: 15882287

[3] Valderhaug TG Hjelmesæth J Hartmann A Røislien J Bergrem HA Leivestad T. The association of early post-transplant glucose levels with long-term mortality. Diabetologia. (2011) 54:1341–9. doi: 10.1007/s00125-011-2105-9, PMID: 21409415 PMC3088823

[4] Porrini E Díaz JM Moreso F Lauzurrica R Ibernon M Torres IS. Prediabetes is a risk factor for cardiovascular disease following renal transplantation. Kidney Int. (2019) 96:1374–80. doi: 10.1016/j.kint.2019.06.026, PMID: 31611066

[5] Valderhaug TG Hjelmesaeth J Rollag H Leivestad T Røislien J Jenssen T. Reduced incidence of new-onset posttransplantation diabetes mellitus during the last decade. Transplantation. (2007) 84:1125–30. doi: 10.1097/01.tp.0000287191.45032.38, PMID: 17998867

[6] Van Laecke S Van Biesen W Verbeke F De Bacquer D Peeters P Vanholder R. Posttransplantation hypomagnesemia and its relation with immunosuppression as predictors of new-onset diabetes after transplantation. Am J Transplant. (2009) 9:2140–9. doi: 10.1111/j.1600-6143.2009.02752.x, PMID: 19624560

[7] Gommers LM Hoenderop JG Bindels RJ de Baaij JH. Hypomagnesemia in type 2 diabetes: a vicious circle? Diabetes. (2016) 65:3–13. doi: 10.2337/db15-1028, PMID: 26696633

[8] MeL L Cruz T Rodrigues LE Bomfim O Melo J Correia R. Serum and intracellular magnesium deficiency in patients with metabolic syndrome—evidences for its relation to insulin resistance. Diabetes Res Clin Pract. (2009) 83:257–62. doi: 10.1016/j.diabres.2008.11.019, PMID: 19124169

[9] Nadler JL Buchanan T Natarajan R Antonipillai I Bergman R Rude R. Magnesium deficiency produces insulin resistance and increased thromboxane synthesis. Hypertension. (1993) 21:1024–9. doi: 10.1161/01.HYP.21.6.1024, PMID: 8505087

[10] Veronese N Dominguez LJ Pizzol D Demurtas J Smith L Barbagallo M. Oral magnesium supplementation for treating glucose metabolism parameters in people with or at risk of diabetes: a systematic review and Meta-analysis of double-blind randomized controlled trials. Nutrients. (2021) 13. doi: 10.3390/nu13114074, PMID: 34836329 PMC8619199

[11] Rodríguez-Morán M Guerrero-Romero F. Insulin secretion is decreased in non-diabetic individuals with hypomagnesaemia. Diabetes Metab Res Rev. (2011) 27:590–6. doi: 10.1002/dmrr.1206, PMID: 21488144

[12] Guerrero-Romero F Rodríguez-Morán M. Magnesium improves the beta-cell function to compensate variation of insulin sensitivity: double-blind, randomized clinical trial. Eur J Clin Investig. (2011) 41:405–10. doi: 10.1111/j.1365-2362.2010.02422.x, PMID: 21241290

[13] Nijenhuis T Hoenderop JG Bindels RJ. Downregulation of ca (2+) and mg (2+) transport proteins in the kidney explains tacrolimus (FK506)-induced hypercalciuria and hypomagnesemia. J Am Soc Nephrol. (2004) 15:549–57. doi: 10.1097/01.ASN.0000113318.56023.B6, PMID: 14978156

[14] Van Laecke S Van Biesen W. Hypomagnesaemia in kidney transplantation. Transplant Rev (Orlando). (2015) 29:154–60. doi: 10.1016/j.trre.2015.05.002 26001746

[15] Lahav I Steinmetz T Molcho M Lev N Agur T Nesher E. The association between exposure to low magnesium blood levels after renal transplantation and cardiovascular morbidity and mortality. Front Med (Lausanne). (2021) 8:690273. doi: 10.3389/fmed.2021.690273, PMID: 34322504 PMC8310919

[16] Panthofer AM Lyu B Astor BC Singh T Aziz F Mandelbrot D. Post-kidney transplant serum magnesium exhibits a U-shaped association with subsequent mortality: an observational cohort study. Transpl Int. (2021) 34:1853–61. doi: 10.1111/tri.13932, PMID: 34081803

[17] Huang JW Famure O Li Y Kim SJ. Hypomagnesemia and the risk of new-onset diabetes mellitus after kidney transplantation. J Am Soc Nephrol. (2016) 27:1793–800. doi: 10.1681/ASN.2015040391, PMID: 26449610 PMC4884111

[18] Van Laecke S Nagler EV Taes Y Van Biesen W Peeters P Vanholder R. The effect of magnesium supplements on early post-transplantation glucose metabolism: a randomized controlled trial. Transpl Int. (2014) 27:895–902. doi: 10.1111/tri.12287, PMID: 24909487

[19] Van Laecke S Caluwe R Huybrechts I Nagler EV Vanholder R Peeters P. Effect of magnesium supplements on insulin secretion after kidney transplantation: a randomized controlled trial. Ann Transplant. (2017) 22:524–31. doi: 10.12659/AOT.903439, PMID: 28848225 PMC12577509

[20] Åsberg A Bjerre A Almaas R Luis-Lima S Robertsen I Salvador CL. Measured GFR by utilizing population pharmacokinetic methods to determine Iohexol clearance. Kidney Int Rep. (2020) 5:189–98. doi: 10.1016/j.ekir.2019.11.012, PMID: 32043033 PMC7000849

[21] Matsuda M DeFronzo RA. Insulin sensitivity indices obtained from oral glucose tolerance testing: comparison with the euglycemic insulin clamp. Diabetes Care. (1999) 22:1462–70. doi: 10.2337/diacare.22.9.1462, PMID: 10480510

[22] Hayashi T Boyko EJ Sato KK McNeely MJ Leonetti DL Kahn SE. Patterns of insulin concentration during the OGTT predict the risk of type 2 diabetes in Japanese Americans. Diabetes Care. (2013) 36:1229–35. doi: 10.2337/dc12-0246, PMID: 23275353 PMC3631850

[23] Hjelmesaeth J Midtvedt K Jenssen T Hartmann A. Insulin resistance after renal transplantation: impact of immunosuppressive and antihypertensive therapy. Diabetes Care. (2001) 24:2121–6. doi: 10.2337/diacare.24.12.2121, PMID: 11723094

[24] Stumvoll M Van Haeften T Fritsche A Gerich J. Oral glucose tolerance test indexes for insulin sensitivity and secretion based on various availabilities of sampling times. Diabetes Care. (2001) 24:796–7. doi: 10.2337/diacare.24.4.796, PMID: 11315860

[25] Oxford TUo. HOMA Calculator. (2018). Available at: https://www.dtu.ox.ac.uk/homacalculator/download.php.

[26] Pacini G Mari A. Methods for clinical assessment of insulin sensitivity and beta-cell function. Best Pract Res Clin Endocrinol Metab. (2003) 17:305–22. doi: 10.1016/S1521-690X(03)00042-3 12962688

[27] Hjelmesaeth J Jenssen T Hagen M Egeland T Hartmann A. Determinants of insulin secretion after renal transplantation. Metabolism. (2003) 52:573–8. doi: 10.1053/meta.2003.50092, PMID: 12759886

[28] Kahn SE Prigeon RL McCulloch DK Boyko EJ Bergman RN Schwartz MW. Quantification of the relationship between insulin sensitivity and beta-cell function in human subjects. Evidence for a hyperbolic function. Diabetes. (1993) 42:1663–72. doi: 10.2337/diab.42.11.1663 8405710

[29] Draznin B Aroda VR Bakris G Benson G Brown FM Freeman R. 2. Classification and diagnosis of diabetes: standards of medical Care in Diabetes-2022. Diabetes Care. (2022) 45:S17–38. doi: 10.2337/dc22-S002, PMID: 34964875

[30] Sharif A Chakkera H de Vries APJ Eller K Guthoff M Haller MC. International consensus on post-transplantation diabetes mellitus. Nephrol Dial Transplant. (2024) 39:531–49. doi: 10.1093/ndt/gfad258, PMID: 38171510 PMC11024828

[31] Nakamura A Iwami D Miyoshi H Morita K Taguri M Terauchi Y. Impact of renal transplantation on glucose tolerance in Japanese recipients with impaired glucose tolerance. Diabet Med. (2017) 34:569–76. doi: 10.1111/dme.13199, PMID: 27505857

[32] Cheungpasitporn W Thongprayoon C Harindhanavudhi T Edmonds PJ Erickson SB. Hypomagnesemia linked to new-onset diabetes mellitus after kidney transplantation: a systematic review and meta-analysis. Endocr Res. (2016) 41:142–7. doi: 10.3109/07435800.2015.1094088, PMID: 26934195

[33] Association AD. Diagnosis and classification of diabetes mellitus. Diabetes Care. (2014) 37:S81–90. doi: 10.2337/dc14-S081, PMID: 24357215

[34] Porrini E Delgado P Alvarez A Cobo M Pérez L González-Posada JM. The combined effect of pre-transplant triglyceride levels and the type of calcineurin inhibitor in predicting the risk of new onset diabetes after renal transplantation. Nephrol Dial Transplant. (2008) 23:1436–41. doi: 10.1093/ndt/gfm762, PMID: 18029372

[35] Jenssen T Hartmann A. Post-transplant diabetes mellitus in patients with solid organ transplants. Nat Rev Endocrinol. (2019) 15:172–88. doi: 10.1038/s41574-018-0137-7 30622369

[36] Porrini EL Díaz JM Moreso F Delgado Mallén PI Silva Torres I Ibernon M. Clinical evolution of post-transplant diabetes mellitus. Nephrol Dial Transplant. (2016) 31:495–505. doi: 10.1093/ndt/gfv368, PMID: 26538615

[37] Barbagallo M Dominguez LJ. Magnesium and type 2 diabetes. World J Diabetes. (2015) 6:1152–7. doi: 10.4239/wjd.v6.i10.1152, PMID: 26322160 PMC4549665

[38] Mooren FC. Magnesium and disturbances in carbohydrate metabolism. Diabetes Obes Metab. (2015) 17:813–23. doi: 10.1111/dom.12492, PMID: 25974209

[39] Gommers LMM Hill TG Ashcroft FM de Baaij JHF. Low extracellular magnesium does not impair glucose-stimulated insulin secretion. PLoS One. (2019) 14:e0217925. doi: 10.1371/journal.pone.0217925, PMID: 31163064 PMC6548430

[40] Hecking M Kainz A Werzowa J Haidinger M Döller D Tura A. Glucose metabolism after renal transplantation. Diabetes Care. (2013) 36:2763–71. doi: 10.2337/dc12-2441, PMID: 23656979 PMC3747896

[41] Reddy ST Soman SS Yee J. Magnesium balance and measurement. Adv Chronic Kidney Dis. (2018) 25:224–9. doi: 10.1053/j.ackd.2018.03.002, PMID: 29793660

[42] DeFronzo RA Tobin JD Andres R. Glucose clamp technique: a method for quantifying insulin secretion and resistance. Am J Physiol Endocrinol Metab. (1979) 237:E214–23. doi: 10.1152/ajpendo.1979.237.3.E214, PMID: 382871

